# Exogenous Calcium Induces Different Hydraulic Strategies in Response to Osmotic Stress in Maize Seedlings

**DOI:** 10.3390/plants12101999

**Published:** 2023-05-16

**Authors:** Dongyang Li, Minfei Yan, Haofeng Liang, Zhe Li, Suiqi Zhang

**Affiliations:** 1 State Key Laboratory of Soil Erosion and Dryland Farming on the Loess Plateau, Northwest A&F University, Yangling 712100, China; 2 College of Life Sciences, Northwest A&F University, Yangling 712100, China; 3 State Key Laboratory of Soil Erosion and Dryland Farming on the Loess Plateau, Institute of Soil and Water Conservation, Chinese Academy of Sciences and Ministry of Water Resources, Yangling 712100, China

**Keywords:** calcium, whole−plant water balance, abscisic acid, root hydraulic conductance, aquaporin, hydrogen peroxide

## Abstract

Recent discoveries regarding the signal molecules involved in abiotic stresses require integration into the field of plant hydraulic property research. Although calcium (Ca) is an important second messenger involved in numerous complex, abiotic stress−induced signaling pathways, it remains unclear how exogenous calcium mediates cellular signaling to promote plant drought resistance. We investigated the effects of calcium on the water balance and hydraulic properties in maize seedlings (*Zea mays* L.) under osmotic stress simulated by 10% (m/v) PEG−6000 in a hydroponic culture. The osmotic stress dramatically decreased the photosynthetic rate, transpiration rate, stomatal conductance, leaf water content, and root water absorption. However, the short−term (2 h) and long−term (10 d) exogenous Ca^2+^ (CaCl_2_: 10 mM) treatments had different effects on the maize gas exchange parameters and leaf water status. The short−term treatment improved the leaf transpiration by inhibiting the abscisic acid (ABA) synthesis and accumulation in the leaves, generating a stronger transpiration pull and enhancing the root water absorption and axial flow path water transport by increasing the root hydraulic conductance to relieve the osmotic stress−induced inhibition. The long−term treatment induced the ABA and H_2_O_2_ accumulation in the roots and leaves. Under osmotic stress, the accumulation of ABA, H_2_O_2_, and Ca^2+^ rapidly repressed the transpiration and enhanced the radial flow path water transport, decreasing the water loss and improving the stress tolerance. These insights suggest a role for a judicious use of Ca fertilizer in reducing the adverse effects of drought on agricultural production.

## 1. Introduction

Calcium (Ca) is not only an essential macronutrient for plant growth and development, but also acts as a universal second messenger in regulating plant responses to biotic and abiotic stresses [1,2,3,4,5]. Abiotic stress can induce spatial and temporal changes in cellular Ca^2+^ concentrations, which are often referred to as the emergence of Ca^2+^ signaling [6]. Ca^2+^ signaling is a vital adaptive strategy in response to developmental cues and external stimuli, such as gravity, nutrient status, mechanical stimulation, temperature shifts, light, salinity, drought, heavy metals, and metalloids [7].

Stress stimuli can be sensed by plant cells and thereby induce specific calcium signatures (the repetitive oscillation or spiking of [Ca^2+^]_cyt_), which are regulated by Ca^2+^ channels, pumps, or carriers localized at the plasma membrane or in the membranes of the organelles [8,9]. In Arabidopsis, OSCA1 family proteins (hyperosmolarity−gated calcium channels) can sense the decreased cellular turgor pressure induced by drought, leading to a Ca^2+^ influx into the guard and root cells [10]. The large families of the downstream Ca^2+^ sensors, including calmodulins (CaMs) and calmodulin−like proteins (CMLs); Ca^2+^−dependent protein kinases (CDPKs) and CDPK−related kinases (CRKs); and calcineurin B−like proteins (CBLs) and CBL−interacting protein kinases (CIPKs), mediate this process. These proteins sense and decode the information communicated by the specific Ca^2+^ signatures via a conserved EF−hand domain that can trigger the target gene expression and altered phosphorylation, leading to physiological responses that correspond to the original stimuli [6,11].

Alongside the Ca^2+^ signaling, abscisic acid (ABA) and the reactive oxygen species (ROS) also act pivotally in response to biotic and abiotic stresses [12,13]. When plants are exposed to drought stress, ABA is synthesized in the leaves or in the roots and transported to the leaves via the xylem transport [14]. The roots sense the water deficit signal and synthesize the CLAVATA3/EMBRYO−SURROUNDING REGION RELATED 25 (CLE25) peptide, which in turn promotes the ABA synthesis in the root and leaf cells [15]. ABA is perceived by the pyrabactin resistance (PYR)/PYR1−like (PYL)/regulatory components of the ABA receptor (RCAR) which inactivate the group A type 2C protein phosphatases (PP2Cs), resulting in the activation of protein kinases from the subclass III plant−specific sucrose nonfermenting 1-related subfamily 2 (SnRK2s) [16]. Many proteins involved in the drought stress response are activated by the SnRK2s via protein phosphorylation. The above three effector families constitute the core components of the signaling pathway, and their members have been shown to mediate several ABA−controlled plant physiological processes, especially in response to abiotic stress [17,18]. The ROS, including singlet oxygen (^1^O_2_), superoxide (O_2_^−^), hydroxyl radical (·OH), and hydrogen peroxide (H_2_O_2_), are also important signaling molecules that regulate the plant growth, development, and stress responses [19]. One of the most crucial consequences of abiotic stress is the disturbance of the equilibrium between the generation of the ROS and antioxidant defense systems, triggering the excessive accumulation of the ROS and inducing oxidative stress in plants [20]. Apart from their damaging activity, the ROS are well known as secondary messengers or signaling molecules and transduce signals to the nucleus through redox reactions using the mitogen−activated protein kinase (MAPK) pathway in a variety of cellular mechanisms to increase the tolerance against diverse abiotic stresses [21,22]. In addition, H_2_O_2_ is a critical component of stress response regulation in crops such as rice, wheat, maize, and soybean, etc. [23]. Research on ABA, ROS, and Ca^2+^ signal transduction has flourished since the identification and characterization of the core signaling components involved in these pathways [24]. However, many such investigations have focused on the aboveground tissues, whereas the function of ABA, ROS, and Ca^2+^ signal transduction in the whole−plant water balance and root−related processes remains poorly understood.

Water flow through plants is a passive mechanism (diffusion and bulk flow). By analogy to an electric circuit and Ohm’s law, it can be described as the movement of water along a hydraulic circuit with resistance. The flux of the water is along the hydraulic circle in the root, shoot, and canopy, otherwise known as the soil–plant–atmosphere continuum (SPAC) system [25]. In this system, the transpiration of the plant aerial parts modulated by the stomata provides the main driving force behind the root water absorption and the whole−plant water transport. The dynamic balance between the water dissipation by the shoots and the water absorption by the roots plays an important role in maintaining the normal physiological processes in plants, such as photosynthesis, respiration, and plant homeostasis [26]. Especially when plants are under drought stress, the ability to maintain a water balance is an important factor in their survival. In order to rapidly adjust to the external and localized water availability in the roots, the roots must respond rapidly to any changes in the transpiration demand by the shoot. The composite model of the water transport in the roots [27] postulates that water moves radially across the roots along two paths: (1) the apoplast path along the cell wall continuum; and (2) the cell−to−cell path, in which water moves from cell to cell along the common symplast through plasmodesmata or crosses cell membranes, either by simple diffusion or by facilitated diffusion through aquaporins (AQPs). The presence of AQPs in cell membranes permits the regulation of the water flux between and within the cells [28].

In contrast to intracellular Ca^2+^, Ca^2+^ can also act as an exogenous first messenger, which is known to be regulated by the calcium sensing receptor (CAS). When exposed to extracellular Ca^2+^, the induced cytoplasmic Ca^2+^([Ca^2+^]_cyt_) oscillation promotes stomatal closure on the guard cells [29]. In Arabidopsis, ${Ca}_{o}^{2+}$ triggers ${Ca}_{i}^{2+}$ transients in the guard cells through the CAS signaling pathway, which further induces the H_2_O_2_ and NO accumulation and ultimately the stomatal closure [30]. Drought induces osmotic stress in plant cells, and drought or osmotic stress−induced damage in plants results from both water scarcity and the imbalance of water loss and absorption. The purpose of this study was to explore how exogenous calcium induces ABA, ROS, and Ca^2+^ signal transduction, and thus elucidate the different hydraulic strategies induced by calcium under osmotic stress conditions.

## 2. Results

### 2.1. Calcium Contents

**Figure 1:**
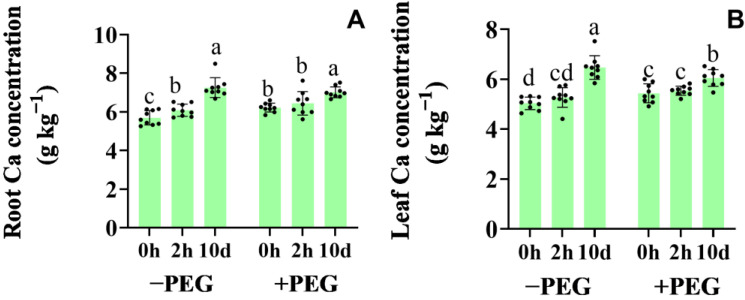
Effects of the calcium application and osmotic stress on the root (**A**) and leaf (**B**) calcium concentration under well−watered conditions (−PEG) and osmotic stress (+PEG) at 2 h (2−h extra calcium treatment) and 10 d (10−d extra calcium treatment). Values are the means ± the SD. Different letters indicate the statistically significant differences (*p* < 0.05) after the ANOVA and Duncan’s test.

The osmotic stress induced a slight increase in the calcium (Ca) contents of the roots and leaves of the maize seedlings. The calcium contents following ten days of exogenous (10−d extra) calcium treatment were 26% higher in the roots, and 28% higher in the shoots than those in the untreated plants under well−watered conditions (Figure 1). However, the increases caused by the 10−d extra calcium treatment still existed under the osmotic stress conditions. Conversely, the 2−h extra calcium treatment caused no change in the calcium contents in the roots and leaves under either set of conditions.

### 2.2. Effect of Calcium on the Photosynthetic Rate, Stomatal Conductance, and Transpiration Rate

**Figure 2:**
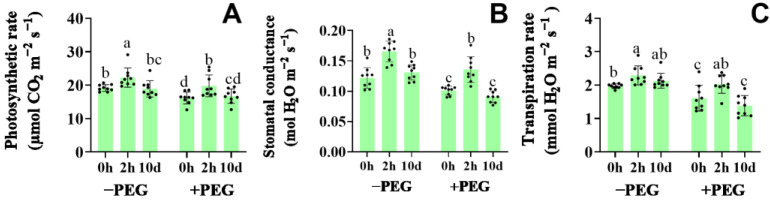
Effects of the calcium application and osmotic stress on photosynthetic rate (**A**), stomatal conductance (**B**), and transpiration rate (**C**) under well−watered conditions (−PEG) and osmotic stress (+PEG) at 2 h (2−h extra calcium treatment) and 10 d (10−d extra calcium treatment). Values are the means ± the SD. Different letters indicate the statistically significant differences (*p* < 0.05) after the ANOVA and Duncan’s test.

Under well−watered conditions, the photosynthetic rate (Pn), stomatal conductance (GS), and transpiration rate (E) of the 2−h extra calcium treatment were, respectively, 16%, 36%, and 16% higher than those of the untreated seedlings (Figure 2). When the plants were exposed to osmotic stress, significant decreases in the Pn, GS, and E were observed. However, these values were ameliorated somewhat by the calcium treatment, and were, respectively, 13%, 19%, and 28% higher in the 2−h extra calcium treatment seedlings compared to the untreated plants. Conversely, the 10−d extra calcium treatment did not significantly affect the Pn, GS, and E under either well−watered or osmotic stress conditions. These results showed that the 2−h extra calcium treatment could improve the Pn, GS, and E under both the well-watered and osmotic stress conditions. Remarkably, osmotic stress significantly decreased the E to suppress the water desorption of the plant shoots. The 10−d extra calcium treatment had no significant effects on the Pn and GS, but slightly suppressed the E of the seedlings under osmotic stress. Thus, the different calcium treatment times exerted different influences on the plant responses to the osmotic stress conditions.

### 2.3. Effect of Calcium on the Leaf Relative Water Content and Leaf Water Potential

**Figure 3:**
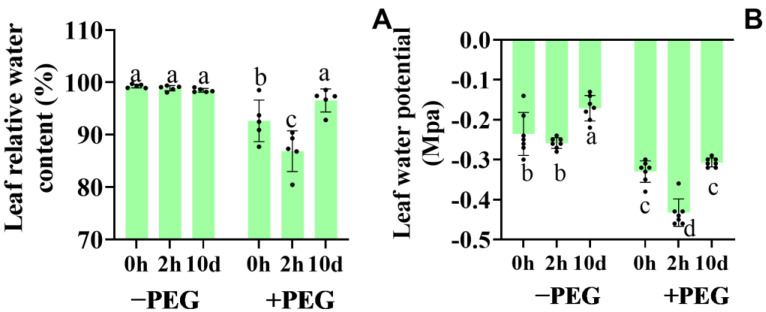
Effects of the calcium application and osmotic stress on the leaf relative water content (RWC) (**A**) and water potential (**B**). Values are the means ± the SD. Different letters indicate the statistically significant differences (*p* < 0.05) after the ANOVA and Duncan’s test.

The leaf relative water content (RWC) and leaf water potential reflect the plant water capacity and the water conservation ability of the leaves, respectively. Under well−watered conditions, the RWC was not affected by the extra calcium treatment (Figure 3A). Conversely, under osmotic stress, the RWC decreased by 6%, and the 2−h extra calcium treatment aggravated this decrease (12%), while the 10−d extra calcium treatment reversed it.

The leaf water potential reflected the leaf water condition of the plants (Figure 3B). Under the well−watered conditions, the 2−h extra calcium treatment did not affect the leaf water potential, whereas it increased by 25% following the 10−d extra calcium treatment. Under osmotic stress, the leaf water potential significantly decreased, but the 10−d extra calcium treatment had no effect. Conversely, the 2−h extra calcium treatment significantly decreased the leaf water potential under the osmotic stress conditions.

These results indicated that the 2−h extra calcium treatment improved the transpiration and preserved the normal photosynthesis under osmotic stress. Conversely, the 10−d extra calcium treatment reduced the water loss and enhanced the plant water conservation capacity under osmotic stress.

### 2.4. Effect of Calcium on the Root Water Uptake and Transport under Osmotic Stress

Under the control conditions, the 2−h extra calcium treatment improved the root hydraulic conductivity (Lpr) slightly, while other treatments had no effect on the Lpr (Table 1). However, under osmotic stress, the 2−h extra calcium treatment ameliorated the stress−induced Lpr decrease. Meanwhile, the 10−d extra calcium treatment slightly suppressed the Lpr under the osmotic stress conditions. The PEG treatment decreased the hydraulic conductance by 27%, while the 2−h extra calcium treatment ameliorated this decrease to only 11%. The 10−d extra calcium treatment caused a 37% decrease in the root hydraulic conductance under the PEG treatment. The 10−d extra calcium treatment also increased the total root surface.

The hydraulic conductivity of the root cortical cells was identified by the cell pressure probe. The cell turgor reflected the cell water condition, which was not affected by the calcium application under the control conditions. The cell turgor declined significantly during osmotic stress but had no response to the calcium treatment (Table 1). The 10−d extra calcium treatment decreased the elastic modulus of the cortex cell wall in the well−watered and stressed plants. The PEG treatment significantly increased the elastic modulus, while the 2−h extra calcium treatment had no obvious influence on the elastic modulus compared to the untreated seedlings. *T_1/2_* (half−time of the water exchange), which reflected the water transmembrane transport time, increased by 60% under osmotic stress. However, *T_1/2_* decreased in the 10−d extra calcium−treated seedlings, especially under osmotic stress, where *T_1/2_* decreased by only 29% compared to the non−calcium−treated seedlings. Moreover, *T_1/2_* was not affected by the 2−h extra calcium treatment under either the control or osmotic stress conditions. The hydraulic conductivity of the cortex cells was not affected by the 2−h extra calcium treatment. A significant decrease in the Lpc was observed when the plants were exposed to osmotic stress, and the 10−d extra calcium treatment increased the Lpc under both well−watered and osmotic stress conditions. These results indicated that the 10−d extra calcium treatment improved the hydraulic conductivity of the root cortical cells to enhance the relative contribution of the cell−to−cell water pathway during osmotic stress.

**Figure 4:**
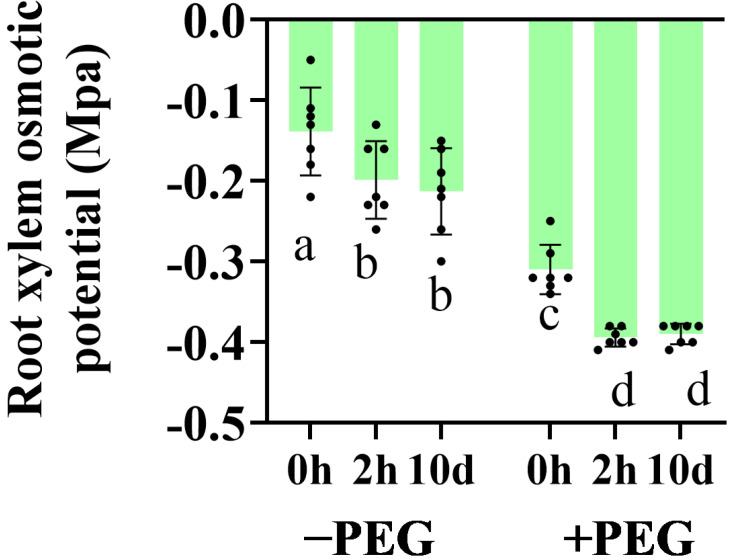
Effects of the calcium application and osmotic stress on the xylem osmotic potential. Values are the means ± the SD. Different letters indicate the statistically significant differences (*p* < 0.05) after the ANOVA and Duncan’s test.

The xylem−exuded sap was collected to measure the xylem osmotic potential. The osmotic potential of the root xylem sap was reduced with the calcium treatment under the control conditions (Figure 4). Under osmotic stress, although the potential of the root xylem sap was decreased, the potential of the calcium−treated seedlings was even lower. These results revealed that calcium improved the ability of the roots to absorb water in response to the osmotic pressure gradient under osmotic stress.

### 2.5. Calcium−Induced ABA Accumulation and Gene Expression of the Core ABA Signaling

**Figure 5:**
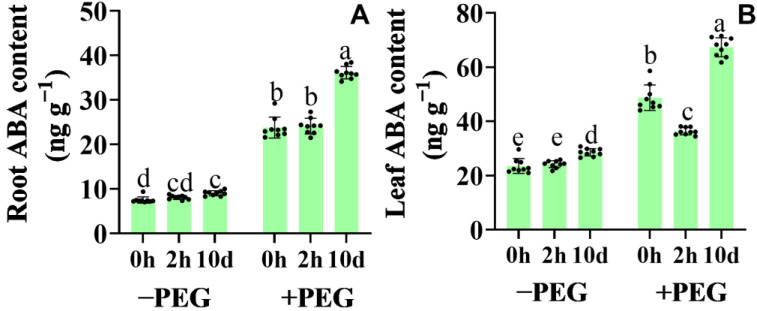
Effects of the calcium application and osmotic stress on the abscisic acid content in the root (**A**) and leaf (**B**). Values are the means ± the SD. Different letters indicate the statistically significant differences (*p* < 0.05) after the ANOVA and Duncan’s test.

Under well−watered conditions, the application of calcium did not affect the ABA content in the seedling roots (Figure 5). Under osmotic stress, however, the ABA content increased in the leaves and roots of the 10−d extra calcium−treated seedlings, whereas the 2−h extra calcium treatment did not affect the ABA content. Interestingly, an obvious decrease was detected in the leaves of the 2−h extra calcium−treated seedlings compared to the non−calcium−treated seedlings under osmotic stress.

*ZmNCED*, *ZmABA3*, *ZmAO3* are the core ABA biosynthesis enzymes. Under the control conditions, the *ZmNCED*, *ZmABA3*, *ZmAO3* expressions did not change significantly after the calcium application (Table 2). After the PEG treatment, *ZmNCED, ZmABA3*, *ZmAO3* were up−regulated and the 10−d extra calcium treatment strongly increased their expression. However, the 2−h extra calcium treatment slightly down−regulated their expression. Three type 2C protein phosphatases (*ZmPP2CA*, *ZmABI1,* and *ZmABI2*) and four protein kinases (*ZmSnRK*2.2, *ZmSnRK*2.3, *ZmSnRK*2.6, and *ZmSnRK*2.10) were selected for further investigation as the core ABA signaling components. Both the *ZmPP2C* and *ZmSnRK2* gene families exhibited distinct expression patterns in response to osmotic stress (or calcium) between the roots and leaves (Table S1).

The levels of the *ZmPP2C* transcripts rose significantly following the 10−d extra calcium treatment under both the control conditions and osmotic stress in the roots and leaves (Table S1). Conversely, the 2−h extra calcium treatment did not affect the *ZmPP2C* expression compared to the non−calcium−treated seedlings. The expressions of *ZmPP2CA*, *ZmABI1,* and *ZmABI2* were significantly up−regulated in the maize roots under osmotic stress. Furthermore, the expression of *ZmABI2* was induced by osmotic stress in the leaves. The *ZmSnRK2* gene families also exhibited an up−regulated expression in response to the 10−d extra calcium treatment under both the control and osmotic stress conditions but showed no response to the 2−h extra calcium treatment. These results revealed that calcium increased the ABA accumulation by up−regulating the expression of the core ABA biosynthesis− and signaling pathway−related genes.

### 2.6. Effect of Calcium on the Hydrogen Peroxide Synthesis and Antioxidant Defense Systems

**Figure 6:**
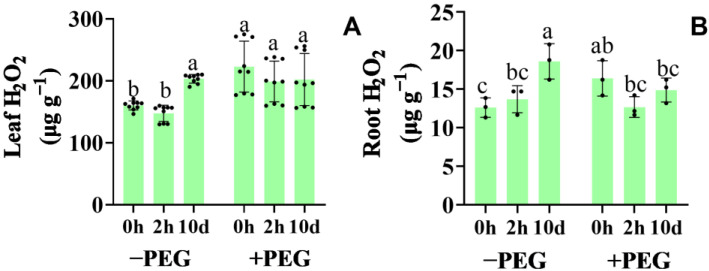
Effects of the calcium application and osmotic stress on the hydrogen peroxide content in the leaf (**A**) and root (**B**). Values are the means ± the SD. Different letters indicate the statistically significant differences (*p* < 0.05) after the ANOVA and Duncan’s test.

The hydrogen peroxide (H_2_O_2_) content increased in the 10−d extra−treated seedling roots and leaves under the control conditions, whereas the 2−h extra calcium treatment did not affect the H_2_O_2_ content (Figure 6). When exposed to osmotic stress, the H_2_O_2_ content increased especially in the leaves, whereas the calcium-treated seedlings exhibited no change, except in the case of the 2−h extra calcium treatment, which caused a slight decrease in the root H_2_O_2_ content under osmotic stress.

The plasma membrane NADPH oxidases (Respiratory Burst Oxidase Homologs [RBOHs]) mediate the apoplastic ROS production in plants. The RBOHs transfer electrons from NADPH to extracellular O_2_ to form O_2_^−^, which is subsequently dismutated to H_2_O_2_. The transcripts of *ZmRBOHs* increased dramatically after the 10−d extra calcium treatment compared to the non-calcium-treated plants, whereas the transcripts of *ZmRBOHs* following the 2−h extra calcium treatment showed no significant changes in either the roots or leaves under the control or osmotic stress conditions (Table S2). Although the transcripts of *ZmRBOHA* and *ZmRBOHC* were up−regulated under osmotic stress, the transcripts of the other *ZmRBOHs* remained stable.

As efficient pathways to modulate the ROS signaling, the activities of CAT, SOD, APX, and GR were enhanced significantly under osmotic stress in the leaf and root (Table 3). Furthermore, the activities of the antioxidant enzymes were enhanced by the calcium treatment, especially the 10−d extra calcium treatment.

To further characterize the antioxidative response to calcium and osmotic stress, the transcription of the antioxidant enzymes was determined. Under the control conditions, the expressions of the antioxidant genes *ZmCAT1*, *ZmSOD4*, *ZmAPX,* and *ZmGR1* were up−regulated by the 10 d calcium treatment, whereas the 2 h calcium treatment had no significant effect (Table S3). When exposed to osmotic stress, the antioxidant gene expression was up−regulated prior to the calcium treatment. The calcium application further elevated the transcript levels of the antioxidant enzymes. Regarding the antioxidant enzyme activities associated with the gene expression, the 10−d extra calcium treatment not only enhanced the antioxidant system, but also increased the transcripts of the *ZmRBOHs* and the regulating ability of the ROS signaling.

### 2.7. Effect of Calcium on the PIPs Expression during the Osmotic Stress Conditions

**Figure 7:**
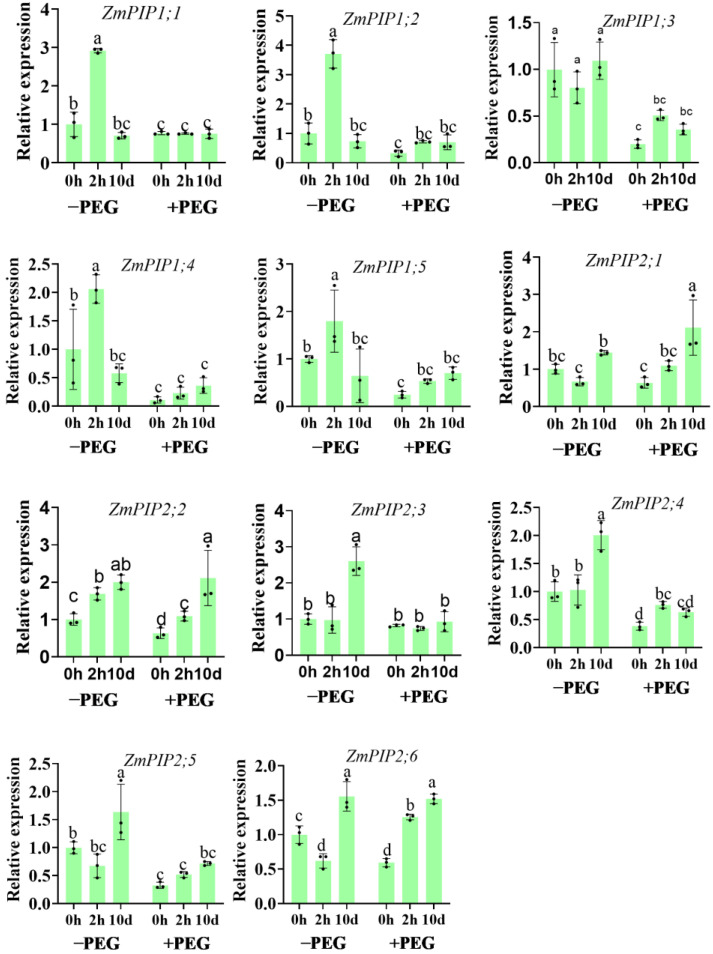
Effects of the calcium application and osmotic stress on the AQP expression in the root. Values are the means ± the SD. Different letters indicate the statistically significant differences (*p* < 0.05) after the ANOVA and Duncan’s test.

The *ZmPIP1s* and *ZmPIP2s* exhibited distinct expression patterns in response to the calcium treatment and osmotic stress between the roots and leaves. In the seedling roots under the control conditions, the expression levels of the *ZmPIP1s* were up−regulated following the 2−h extra calcium treatment but exhibited no change following the 10−d extra calcium treatment (Figure 7). Otherwise, in the seedling leaves, the 2−h extra calcium treatment up-regulated *ZmPIP1;2* and *ZmPIP1;4* under the control conditions (Table S4). The expression of the *ZmPIP1s* decreased in the leaves under osmotic stress, and this decrease was ameliorated by the calcium application. When exposed to osmotic stress, the expression levels of the *ZmPIP1s* in the roots were down-regulated, with the exception of *ZmPIP1;1*, but up-regulated by the 2 h or 10−d extra calcium treatments. The expression levels of the *ZmPIP2s* in the roots were up−regulated by the 10−d extra calcium treatment and were down-regulated by the 2−h extra calcium treatment compared to the untreated plants under the control conditions. After 2 h of osmotic stress, the expression levels of the *ZmPIP2s* were up-regulated by the calcium application, especially significantly following the 10−d extra calcium treatment.

## 3. Discussion

Plants have evolved three strategies to adapt to drought, namely, drought escape, drought tolerance, and drought avoidance. Drought escape refers to the acceleration of a plant’s life cycle before stress affects its survival [31]. Drought tolerance refers to maintaining growth with low water content over the drought period through osmotic adjustment, reactive oxygen species (ROS) scavenging, and the activation of stress−related genes [32]. Drought avoidance refers to reducing water loss through fast stomatal closure and long−term growth inhibition until the next chance for water uptake. In our study, the 2−h extra calcium and 10−d extra calcium treatments activated different strategies under osmotic stress. The 2 h extra calcium treatment improved photosynthesis and transpiration to relieve the osmotic stress−induced inhibition of these processes. On the other hand, the 10−d extra calcium treatment reduced transpiration and stomatal conductance in order to reduce water loss, thereby maintaining a relatively high water level for resisting osmotic stress. The different strategies carried out were both reasonable and suitable for the adaptation of the plants to osmotic stress. In order to clarify why the plants carried out different strategies following the calcium application under osmotic stress, we focused on the signaling transduction of the ABA, ROS, and Ca^2+^ induced by the exogenous calcium. The 10−d extra calcium treatment induced the Ca^2+^ accumulation in the leaves and roots, while the 2−h extra calcium did not affect the calcium content, and a higher calcium content induced the up−regulated transcription of the core ABA biosynthesis enzymes and the ABA accumulation in the leaves. Plants can also sense water deficit conditions at their roots and transmit these signals to their shoots to synthesize ABA in their leaves [15]. Under osmotic stress, the leaf ABA content increased following the treatment with 10−d of extra calcium, whereas the 2−h extra calcium treatment decreased the leaf ABA content. Meanwhile, the 2−h extra calcium treatment markedly down−regulated the expression of the core ABA biosynthesis enzymes. Playing a key determinative role in regulating stomatal closure under drought stress, ABA can be rapidly induced water deficit signals to promote stomatal closure via the regulation of the downstream signaling components [18]. The ROS also played a key role in controlling stomatal closure in response to the water deficit stress. The ROS are known to participate in the ABA−mediated stomatal closure. The production and accumulation of apoplastic ROS depend on the ABA signaling, suggesting that both ABA and the ROS are important for stomatal closure [19]. However, whereas the 10−d extra calcium treatment induced the elevated leaf H_2_O_2_ content under the control conditions, the calcium treatment did not affect the leaf H_2_O_2_ content under osmotic stress. In addition, the Ca^2+^−induced stomatal closure was regulated by ABA and ROS, which increased the concentration of cytosolic Ca^2+^ in the guard cells [24]. Several ABA signaling components are regulated by Ca^2+^−mediated signaling. As a result, the 10−d extra calcium treatment−enhanced ABA signaling helped the plants improve drought resistance and survive in a water deficit stress environment by reducing water loss. ABA is perceived using pyrabactin resistance (PYR)/PYR1−like (PYL)/regulatory components of ABA receptors (RCARs), which inactivate PP2C, resulting in the activation of SnRK2 protein kinases [16]. ABA stimulated by calcium, together with calcium signaling, enhanced the ABA signaling pathway. However, the 2−h extra calcium treatment reduced the ABA content in the leaves, thereby improving the transpiration to maintain the normal physiology process.

The water balance helps control the water content and improve the plant survivability during drought, which includes reducing shoot water loss and increasing root water absorption. So, the regulation of the stomata closure and root water absorption is an efficient way for plants to reduce water loss during drought. Many previous studies discussed the important role of the stomata in plant regulation of photosynthesis and transpiration under drought stress. However, fewer studies investigated the influence of root water absorption under drought stress. Water uptake through the root system can be increased by three basic means: (1) an increase in the absorbing surface area; (2) an increase in intrinsic water uptake properties (hydraulic conductivity) per unit surface and driving force (water potential gradient); and (3) an increase in the biophysical force that drives the water uptake between the root medium and the xylem [25]. The Lpr of an individual root depends on the hydraulic properties of the axial flow path along (predominantly) mature metaxylem vessels and the radial flow path between the root epidermis and stele. It is generally accepted that the radial flow path constitutes the hydraulic bottleneck within the roots and is the limiting factor in the hydraulic conductance [33]. Under osmotic stress, the 2−h extra calcium treatment improved the root hydraulic conductance, which slightly decreased after the 10−d extra calcium treatment. The 2−h extra calcium treatment enhanced the water axial flow path transport compared to the 10−d extra calcium treatment, indicating that the 2−h extra calcium−treated plants exerted a more powerful transpiration pull. 

The radial flow paths consist of two water transport routes arranged in parallel: the apoplastic and cell−to−cell paths. The apoplastic path provides free diffusional space outside the plasma membrane and consists mainly of the cell wall space. The cell−to−cell path involves the symplastic flow through the plasmodesmata and the transmembrane flow across the cellular membranes. It is currently not possible to distinguish experimentally between the symplastic and transmembrane paths. The gradients in the hydrostatic pressure between the root medium and xylem can drive the radial water uptake in two ways. Along the cell−to−cell path, a hydrostatic pressure gradient drives the water uptake by the root. Under osmotic stress, the root xylem osmotic penitential was lower in the calcium−treated seedlings, which enhanced the root water transport driven by osmotic pressure [34]. Meanwhile, the 10−d extra calcium treatment improved the hydraulic conductivity of the root cortical cells, which contributed to the cell−to−cell water transport during osmotic stress. Aquaporins, which are gated water−conducting channels, are involved in water transport along the cell−to−cell pathway. The application of calcium, especially the 10−d extra calcium treatment, up−regulated the levels of the *ZmPIP* transcripts under the control and osmotic stress conditions.

## 4. Materials and Methods

### 4.1. Plant Material and Experimental Design

These experiments were conducted in plant growth climatic chambers (TPC7063H; Hangzhou Qiushi Artificial Environment Co. Ltd., Hangzhou, China) with day/night simulation under the following settings: temperature 25/20 °C, relative humidity 50/60%, light/dark 14/10 h, and photon flux density of 400 μmol m^−2^ s^−1^. The maize seeds [*Zea mays* L. cv Zhengdan958] were germinated in a dark incubator at 28 °C for 3 days after sterilization with NaClO (1%). The uniform germinated seeds were selected and transplanted into the hydroponic culture in a plastic container (38 cm × 28 cm × 15 cm).

For the 10−day CaCl_2_ treatments, the maize seedlings were transplanted into a Hoagland nutrient solution containing 100 mM CaCl_2_ and the maize seedlings were grown there for 10 days. Then, osmotic stress was induced by adding 10% PEG6000 (*W*/*V*). The samples were taken 2 h after the PEG treatment.

For the 2−h CaCl_2_ treatments, the maize seedlings were grown in a Hoagland nutrient solution for 10 days, then 100 mM CaCl_2_ and 10% PEG6000 (*w*/*v*) were added. The samples were taken 2 h after the treatment.

The experiments were repeated three times, and each repetition comprised at least three biological replicates.

### 4.2. Ca Concentration

The Ca content in the root and shoot was analyzed using atomic absorption spectrophotometry. A total of 1.0 g of the dried samples was dry−ashed in crucibles. A measure of 20 mL of 1.2 M hydrochloric acid was added, and the mixture was filtered. Then, 1.0 mL of the filtrate and 1.0 mL of LaCl_3_ were diluted and combined into a 50 mL volume, and the Ca content was determined using an atomic absorption spectrophotometer (ZL5100, PerkinElmer Inc., Wellesley, MA, USA).

### 4.3. Photosynthetic Rate, Stomatal Conductance, and Transpiration Rate

The photosynthetic rate, stomatal conductance, and transpiration rate were measured using a portable photosynthesis system (Li−6400; LI−COR Inc., Lincoln, NE, USA). The newest fully expanded leaf was placed in a leaf chamber under a 600 μmol m^−2^ s^−1^ photon flux density, 60% RH (relative humidity), and 400 μmol mol^−1^ CO_2_. Each treatment was performed in six replications.

### 4.4. Leaf Relative Water Content and Water Potential

The leaf relative water content was measured according to Machado [34]. The fully expanded leaf was weighed immediately for the fresh weight (g) (FW). The weighed leaf was soaked in distilled water for 8 h, and then the total weight (g) (TW) was measured. The dry weight (g) (DW) was measured after drying at under 80 °C for 24 h. The leaf relative water content was calculated as follows.

RWC = [(FW − DW)/(TW − DW)] × 100%

The leaf water potential was measured using a pressure chamber (Model 3500; Soil Moisture Equipment Crop., Santa Barbara, CA, USA). Each treatment was performed in six replications.

### 4.5. Root Hydraulic Conductivity (Lpr)

The root hydraulic conductivity (Lpr) was measured using a pressure chamber (Model 3005F01; Soil Moisture Equipment Corp., Santa Barbara, CA, USA). The whole root system was detached from the shoot and 3 cm of the mesocotyl was left to insert into the silicone seals of the pressure chamber. The cylindrical pressure chamber contained a half−strength Hoagland solution, or a PEG−treated nutrient solution based on the different osmotic status. The exuded sap was collected at the pressures of 0.1, 0.2, 0.3, 0.4, and 0.5 Mpa for 60 s, and the sap weight was measured. After the exuded sap was measured, the whole root surface area was determined using a scanner and WinRHIZO PRO 2009 (WinRHIZO, Regent Instrument Inc., Québec, QC, Canada). The Lpr value was calculated according to the following equation.

Lpr = V × S^−1^ × P^−1^ × t^−1^

where Lpr is the root hydraulic conductivity (m s^−1^ MPa^−1^), V is the total volume of the water passing through the root (m^3^), S is the root surface area (m^2^), P is the external pressure (MPa), and t is the time (s).

### 4.6. Osmotic Potential of Root Xylem Sap

The whole root system was detached from the shoot and 3 cm of the mesocotyl was left to insert into the silicone seals of the pressure chamber. The mesocotyl was sealed with silicone seals which had a hole adjusted to the diameter of the mesocotyl. The xylem sap was force−exuded using N_2_ pressurized to two bar. Approx. 15 μL of the root xylem sap was collected and sealed in a microtube. The osmotic potential of the collected sap was determined using a vapor pressure osmometer (Model 5520, Wescor, Logan, UT, USA). Each treatment was performed in six replications.

### 4.7. Hydraulic Conductivity of Root Cortical Cells (Lpc)

The hydraulic conductivity of the root cortical cells (Lpc) was measured using the cell pressure probe. The measurements were performed on the 4th to 6th cell layers of the intact primary root cortical cells at 5–7 cm from the root tip. The cell elastic modulus (ε) and Lpc were determined and calculated as previously described [34]. The hydrostatic pressure relaxations were induced to determine the *T_1/2_* across the cell membranes, which was a measure of the water transport properties of cells: the shorter the *T_1/2_* value, the more permeable the membrane was to water. Together with the values of the elastic modulus, this made it possible to calculate the Lpc.

### 4.8. Hydrogen Peroxide Content

The hydrogen peroxide content was measured according to the method of Yin et al. [35]. The plant tissues (0.5 g) were homogenized with 5 mL of cold 0.1% TCA on ice. The homogenate was centrifuged at 12,000 *g* for 20 min. A measure of 0.5 mL of the supernatant was added to 0.5 mL of 10 mM potassium phosphate buffer (pH 7.0) and 1 mL of 1 M KI. The absorbance of the mixture was read at 390 nm, and the H_2_O_2_ content was calculated according to the standard curve.

### 4.9. Antioxidant Enzyme Activities

The plant tissues (0.5 g) were homogenized using 50 mM of a sodium phosphate buffer (pH 7.8). The homogenate was centrifuged at 15,000× *g* for 20 min, and the supernatants were used to measure the enzyme activity. The superoxide dismutase (SOD) activity was determined by its reaction to nitro blue tetrazolium (NBT) at 560 nm. The catalase (CAT) activity was assayed by testing the rate of the H_2_O_2_ degradation at 240 nm. The ascorbate peroxidase (APX) activity was determined by the oxidized ascorbate, which caused an absorbance decrease at 290 nm.

### 4.10. RNA Purification and Expression Analysis

The total RNA was extracted using a Trizon reagent (TransGen, Beijing, China), and the extracted RNA was treated with recombinant DNase I (RNase−free; Takara Bio, Shiga, Japan) for purification. The single−stranded cDNA was then synthesized using an iScript™ cDNA Synthesis Kit (Bio−Rad, Hercules, CA, USA). The iQ™ SYBR^®^ Green Supermix (Bio−Rad) was used for the qRT−PCR on a MiniOpticon™ CFD−3120J1 instrument (Bio−Rad). The data acquisition and analysis of a qRT−PCR was conducted using the Bio−Rad CFX manager software (version 2.0), and the expression levels of the target genes were normalized to that of the internal control gene Actin1 using the −2^ΔΔCt^ method. The primers are shown in Table S5. Each treatment included three replications and each replication included two technical replications.

### 4.11. ABA Content

A 1.0 g powdered sample (fresh weight) was suspended in 8 mL of 80% (*v*/*v*) methanol containing 200 mg L^−1^ of butylated hydroxytoluene and 500 mg L^−1^ of citric acid monohydrate on ice. The mixture remained stationary overnight at 4 °C before centrifugation for 15 min at 10,000 rpm at 4 °C. The supernatant was subsequently collected, and the precipitate was extracted again for 2 h. The supernatants were then combined, dried under N_2_ and resuspended in 900 μL of 80% methanol. After filtering the samples through a 0.45 μm filter, the ABA concentration in the extracts was analyzed using an LC−20AT high performance liquid chromatography system (Shimadzu, Kinh Do, Japan) and an API 2000™ electrospray tandem mass spectrometer (Allen−Bradley, Milwaukee, WI, USA). The (±)−ABA (product number: A1049, Sigma, St. Louis, MO, USA) was used for the preparation of the standard curves to quantify the hormone concentrations in the samples.

### 4.12. Statistical Analysis

The data were checked for normal distribution and equal variance, then analyzed using a one−way ANOVA and Duncan’s multiple range tests. The interactions were considered significant at *p* < 0.05 using SPSS v.14.0 (SPSS Inc., Chicago, IL, USA). The correlations were examined using Pearson’s correlation coefficient. The SigmaPlot v. 12.5 (Systat Inc., San Jose, CA, USA) was used to correlate and plot the indicators.

## 5. Conclusions

Considering both the shoot water loss and root water absorption, we investigated the different drought−resistance strategies induced by calcium in maize. The experimental results revealed that (1) the short−term calcium treatment improved leaf transpiration by inhibiting the synthesis and accumulation of ABA in the leaves, generating a stronger transpiration pull and enhancing the root water absorption and water transport of the axial flow path in order to relieve the osmotic stress−induced inhibition. (2) The long−term calcium treatment induced the accumulation of ABA and H_2_O_2_. When suffering osmotic stress, the accumulated ABA, together with H_2_O_2_ and Ca^2+^, rapidly induced the repression of the transpiration and strength of the radial flow path water transport in order to decrease the water loss and improve drought resistance. This study revealed that calcium induced different hydraulic strategies in response to osmotic stress, through ABA, ROS, and Ca^2+^ signal transduction, thereby controlling the coordination of the root water absorption and shoot water desorption. These findings suggest a role for Ca fertilizer in reducing the adverse effects of drought on agricultural production.

## Figures and Tables

**Table 1:** Hydraulic conductivity of the entire root and the root cortical cells under osmotic stress.

Treatment		−PEG			+PEG	
	0 h Calcium	2 h Calcium	10 d Calcium	0 h Calcium	2 h Calcium	10 d Calcium
Lpr	18.27 ± 1.28 b	19.94 ± 057 a	18.76 ± 0.88 b	13.25 ± 0.43 d	16.24 ± 0.51 c	11.4 ± 0.73 e
Turgor	0.52 ± 0.022 a	0.53 ± 0.020 a	0.54 ± 0.018 a	0.33 ± 0.009 b	0.35 ± 0.008 b	0.36 ± 0.009 b
ε	0.65 ± 0.022 a	0.70 ± 0.018 a	0.55 ± 0.018 b	1.29 ± 0.077 c	1.23 ± 0.043 cd	1.08 ± 0.026 d
*T_1/2_*	2.41 ± 0.031 c	2.53 ± 0.093 c	2.23 ± 0.052 d	3.93 ± 0.058 a	3.9 ± 0.15 a	3.16 ± 0.17 b
Lpc	22.56 ± 1.28 c	23.7 ± 0.57 b	25.98 ± 0.88 a	9.68 ± 0.43 f	9.34 ± 0.51 e	15.28 ± 0.74 d

Note: the root hydraulic conductivity (Lpr) (10^−7^ m s^−1^ MPa^−1^); turgor (cell turgor) (Mpa); ε/(elastic modulus of the cortex cell wall) (Mpa); *T_1/2_* (half−time of the water exchange) (s); hydraulic conductivity of the cortex cells (Lpc) (10^−7^ m s^−1^ MPa^−1^). Different letters indicate the statistically significant differences (*p* < 0.05) after the ANOVA and Duncan’s test.

**Table 2:** Relative expression of the ABA synthesis enzymes under osmotic stress.

Treatment			−PEG			+PEG	
		0 h Calcium	2 h Calcium	10 d Calcium	0 h Calcium	2 h Calcium	10 d Calcium
	*ZmNCED*	1.00 ± 0.08 c	1.10 ± 0.11 c	1.51 ± 0.14 c	3.47 ± 0.07 a	2.18 ± 0.08 b	3.68 ± 0.11 a
Leaf	*ZmABA3*	1.00 ± 0.27 c	0.92 ± 0.10 c	1.60 ± 0.12 c	3.09 ± 0.12 b	1.82 ± 0.07 c	6.03 ± 0.44 a
	*ZmAO2*	1.00 ± 0.20 c	0.55 ± 0.073 c	0.68 ± 0.045 c	3.27 ± 0.12 b	1.21 ± 0.066 c	5.99 ± 0.12 a
	*ZmNCED*	1.00 ± 0.019 e	0.99 ± 0.008 e	1.54 ± 0.031 d	2.17 ± 0.026 c	2.37 ± 0.031 b	3.12 ± 0.040 a
Root	*ZmABA3*	1.00 ± 0.007 d	0.78 ± 0.042 e	1.38 ± 0.020 c	1.55 ± 0.032 b	1.33 ± 0.038 c	3.10 ± 0.053 a
	*ZmAO2*	1.00 ± 0.002 d	1.00 ± 0.017 d	1.00 ± 0.003 d	2.35 ± 0.056 b	2.13 ± 0.029 c	3.35 ± 0.029 a

Note: Different letters indicate the statistically significant differences (*p* < 0.05) after the ANOVA and Duncan’s test.

**Table 3:** Activity of the antioxidant enzyme during osmotic stress.

Treatment			−PEG			+PEG	
		0 h Calcium	2 h Calcium	10 d Calcium	0 h Calcium	2 h Calcium	10 d Calcium
	*CAT*	38.89 ± 0.43 cd	37.64 ± 1.08 d	40.21 ± 0.89 c	42.58 ± 0.79 b	43.96 ± 0.95 b	51.65 ± 0.66 a
Leaf	*SOD*	41.63 ± 0.69 d	44.78 ± 0.96 c	62.13 ± 2.01 a	40.74 ± 1.76 e	35.81 ± 2.28 f	55.78 ± 0.81 b
	*APX*	0.34 ± 0.004 e	0.35 ± 0.003 de	0.42 ± 0.009 c	0.37 ± 0.013 d	0.47 ± 0.006 b	0.53 ± 0.007 a
	*GR*	19.34 ± 0.41 d	20.65 ± 0.36 c	22.44 ± 0.63 b	24.29 ± 0.43 a	20.31 ± 0.35 cd	21.57 ± 0.52 bc
	*CAT*	5.33 ± 0.146 d	6.78 ± 0.068 c	6.84 ± 0.127 c	7.65 ± 0.245 b	8.07 ± 0.173 b	11.12 ± 0.166 a
Root	*SOD*	33.00 ± 0.59 d	32.23 ± 0.63 d	36.13 ± 0.68 b	35.21 ± 0.57 bc	34.18 ± 0.36 cd	38.95 ± 0.53 a
	*APX*	0.29 ± 0.008 f	0.48 ± 0.008 e	0.64 ± 0.017 d	0.82 ± 0.014 c	1.03 ± 0.021 b	1.13 ± 0.027 a
	*GR*	8.78 ± 0.13 d	9.36 ± 0.39 cd	10.12 ± 0.50 bc	9.23 ± 0.22 d	10.34 ± 0.23 b	11.33 ± 0.24 a

Note: Different letters indicate the statistically significant differences (*p* < 0.05) after the ANOVA and Duncan’s test.

## Data Availability

The data that support the findings of this study are available in the main text and the Supplementary Materials.

## Supplementary Materials

The following supporting information can be downloaded at: https://www.mdpi.com/article/10.3390/plants12101999/s1, Table S1. Relative expression of the core ABA signaling components during osmotic stress; Table S2. Relative expression of *ZmRBOHs* during osmotic stress; Table S3. Relative expression of the antioxidant enzyme during osmotic stress; Table S4. Relative expression of *ZmPIPs* during osmotic stress in the leaves; Table S5. The primers used in the qRT−PCR.

## References

[1] Aldon D., Mbengue M., Mazars C., Galaud J.P. (2018). Calcium Signalling in Plant Biotic Interactions. Int. J. Mol. Sci..

[2] Lee H.J., Seo P.J. (2021). Ca^2+^ talyzing Initial Responses to Environmental Stresses. Trends Plant Sci..

[3] McAinsh M.R., Pittman J.K. (2009). Shaping the calcium signature. New Phytol..

[4] Kudla J., Becker D., Grill E., Hedrich R., Hippler M., Kummer U., Parniske M., Romeis T., Schumacher K. (2018). Advances and current challenges in calcium signaling. New Phytol..

[5] Xiong L., Schumaker K.S., Zhu J.K. (2002). Cell signaling during cold, drought, and salt stress. Plant Cell.

[6] Kudla J., Batistic O., Hashimoto K. (2010). Calcium signals: The lead currency of plant information processing. Plant Cell.

[7] Tong T., Li Q., Jiang W., Chen G., Xue D., Deng F., Zeng F., Chen Z.H. (2021). Molecular Evolution of Calcium Signaling and Transport in Plant Adaptation to Abiotic Stress. Int. J. Mol. Sci..

[8] Allen G.J., Chu S.P., Schumacher K., Shimazaki C.T., Vafeados D., Kemper A., Hawke S.D., Tallman G., Tsien R.Y., Harper J.F. (2000). Alteration of stimulus−specific guard cell calcium oscillations and stomatal closing in Arabidopsis det3 mutant. Science.

[9] Harper J.F. (2001). Dissecting calcium oscillators in plant cells. Trends Plant Sci..

[10] Yuan F., Yang H., Xue Y., Kong D., Ye R., Li C., Zhang J., Theprungsirikul L., Shrift T., Krichilsky B. (2014). OSCA1 mediates osmotic−stress−evoked Ca2+ increases vital for osmosensing in Arabidopsis. Nature.

[11] Dong Q., Wallrad L., Almutairi B.O., Kudla J. (2022). Ca^2+^ signaling in plant responses to abiotic stresses. J Integr. Plant Biol..

[12] Kollist H., Zandalinas S.I., Sengupta S., Nuhkat M., Kangasjärvi J., Mittler R. (2019). Rapid Responses to Abiotic Stress: Priming the Landscape for the Signal Transduction Network. Trends Plant Sci..

[13] Gong Z., Xiong L., Shi H., Yang S., Herrera−Estrella L.R., Xu G., Chao D.Y., Li J., Wang P.Y., Qin F. (2020). Plant abiotic stress response and nutrient use efficiency. Sci. China Life Sci..

[14] Ma Y., Cao J., He J., Chen Q., Li X., Yang Y. (2018). Molecular Mechanism for the Regulation of ABA Homeostasis During Plant Development and Stress Responses. Int. J. Mol. Sci..

[15] Takahashi F., Suzuki T., Osakabe Y., Betsuyaku S., Kondo Y., Dohmae N., Fukuda H., Yamaguchi−Shinozaki K., Shinozaki K. (2018). A small peptide modulates stomatal control via abscisic acid in long−distance signalling. Nature.

[16] Zhu J.K. (2016). Abiotic Stress Signaling and Responses in Plants. Cell.

[17] Fujii H., Chinnusamy V., Rodrigues A., Rubio S., Antoni R., Park S.Y., Cutler S.R., Sheen J., Rodriguez P.L., Zhu J.K. (2009). In vitro reconstitution of an abscisic acid signalling pathway. Nature.

[18] Fujii H., Verslues P.E., Zhu J.K. (2011). Arabidopsis decuple mutant reveals the importance of SnRK2 kinases in osmotic stress responses in vivo. Proc. Natl. Acad. Sci. USA.

[19] Postiglione A.E., Muday G.K. (2020). The Role of ROS Homeostasis in ABA−Induced Guard Cell Signaling. Front. Plant Sci..

[20] Cruz de Carvalho M.H. (2008). Drought stress and reactive oxygen species: Production, scavenging and signaling. Plant Signal. Behav..

[21] Sierla M., Waszczak C., Vahisalu T., Kangasjärvi J. (2016). Reactive Oxygen Species in the Regulation of Stomatal Movements. Plant Physiol..

[22] Singh R., Parihar P., Singh S., Mishra R.K., Singh V.P., Prasad S.M. (2017). Reactive oxygen species signaling and stomatal movement: Current updates and future perspectives. Redox Biol..

[23] Gechev T., Petrov V. (2020). Reactive Oxygen Species and Abiotic Stress in Plants. Int. J. Mol. Sci..

[24] Liu H., Song S., Zhang H., Li Y., Niu L., Zhang J., Wang W. (2022). Signaling Transduction of ABA, ROS, and Ca(2+) in Plant Stomatal Closure in Response to Drought. Int. J. Mol. Sci..

[25] Suku S., Knipfer T., Fricke W. (2014). Do root hydraulic properties change during the early vegetative stage of plant development in barley (Hordeum vulgare)?. Ann. Bot..

[26] Martínez−Vilalta J., Garcia−Forner N. (2017). Water potential regulation, stomatal behaviour and hydraulic transport under drought: Deconstructing the iso/anisohydric concept. Plant Cell Environ..

[27] Steudle E. (2001). The Cohesion−Tension Mechanism and the Acquisition of Water by Plant Roots. Annu. Rev. Plant Physiol. Plant Mol. Biol..

[28] Hachez C., Besserer A., Chevalier A.S., Chaumont F. (2013). Insights into plant plasma membrane aquaporin trafficking. Trends Plant Sci..

[29] Wang W.H., Zheng H.L. (2012). Mechanisms for calcium sensing receptor−regulated stomatal closure in response to the extracellular calcium signal. Plant Signal. Behav..

[30] Wang W.H., Yi X.Q., Han A.D., Liu T.W., Chen J., Wu F.H., Dong X.J., He J.X., Pei Z.M., Zheng H.L. (2012). Calcium−sensing receptor regulates stomatal closure through hydrogen peroxide and nitric oxide in response to extracellular calcium in Arabidopsis. J. Exp. Bot..

[31] Kooyers N.J. (2015). The evolution of drought escape and avoidance in natural herbaceous populations. Plant Sci..

[32] Rane J., Singh A.K., Tiwari M., Prasad P.V., Jagadish S.V. (2021). Effective Use of Water in Crop Plants in Dryland Agriculture: Implications of Reactive Oxygen Species and Antioxidative System. Front. Plant Sci..

[33] Hachez C., Veselov D., Ye Q., Reinhardt H., Knipfer T., Fricke W., Chaumont F. (2012). Short−term control of maize cell and root water permeability through plasma membrane aquaporin isoforms. Plant Cell Environ..

[34] Wang W., Yang X., Zhang S., Sun Y. (2013). The root cortex cell hydraulic conductivity is enhanced with increasing chromosome ploidy in wheat. Plant Physiol. Biochem..

[35] Yin L., Wang S., Eltayeb A.E., Uddin M.I., Yamamoto Y., Tsuji W., Takeuchi Y., Tanaka K. (2010). Overexpression of dehydroascorbate reductase, but not monodehydroascorbate reductase, confers tolerance to aluminum stress in transgenic tobacco. Planta.

