# Supplementary material for: Exogenous Calcium Induces Different Hydraulic Strategies in Response to Osmotic Stress in Maize Seedlings
Source: Plants (Basel). 2023 May 16;12(10):1999. doi: 10.3390/plants12101999 (PMC10223354; doi:10.3390/plants12101999)
Supplement: Supplementary file 1 [file plants-12-01999-s001.zip › plants-2323496-supplementary.pdf]

plants MDPI

Table S1. Relative expression of core ABA signaling components during osmotic stress.

Treatment		-PEG			+PEG		
		0h Calcium	2h Calcium	10d Calcium	0h Calcium	2h Calcium	10d Calcium
Leaf	*ZmABI1*	1.00±0.085cd	1.10±0.068c	1.37±0.081b	0.88±0.082d	1.07±0.083c	1.73±0.097a
	ZmABI2	1.00±0.267e	1.15±0.136e	3.05±0.086d	5.63±0.318a	5.06±0.440c	6.64±0.245b
	ZmPP2CA	1.00±0.006c	1.16±0.031b	1.23±0.067ab	1.19±0.039ab	1.28±0.036a	1.19±0.016ab
	ZmSnRK2.2	1.00±0.24b	0.78±0.045b	0.87±0.048b	0.85±0.070b	0.77±0.154b	2.01±0.069a
	ZmSnRK2.3	1.00±0.11c	1.01±0.046c	2.14±0.070c	7.47±0.98b	6.21±1.026b	22.35±2.205a
	ZmSnRK2.6	1.00±0.086c	0.83±0.071c	1.06±0.108c	2.29±0.393b	2.24±0.341b	7.74±1.373a
	ZmSnRK2.10	1.00±0.103b	0.90±0.114b	1.39±0.138b	1.49±0.143b	1.04±0.140b	2.64±0.205a
Root	*ZmABI1*	1.00±0.005e	1.27±0.033d	1.56±0.032bc	1.54±0.021c	1.64±0.037b	2.30±0.033a
	ZmABI2	1.00±0.004e	0.95±0.030e	3.25±0.087b	1.87±0.055c	1.64±0.043d	4.20±0.094a
	ZmPP2CA	1.00±0.007e	1.00±0.005e	1.17±0.036d	3.05±0.027b	2.77±0.069c	4.63±0.090a
	ZmSnRK2.2	1.00±0.004e	1.26±0.080e	3.95±0.030d	6.41±0.198b	5.61±0.059c	10.48±0.280a
	ZmSnRK2.3	1.00±0.004e	0.947±0.030e	3.25±0.087b	1.87±0.055c	1.64±0.043d	4.20±0.094a
	ZmSnRK2.6	1.00±0.004e	0.81±0.0048f	2.42±0.014d	5.61±0.058b	3.20±0.046c	7.59±0.046c
	ZmSnRK2.10	1.00±0.004d	0.92±0.018d	1.22±0.011c	1.59±0.033b	1.16±0.029c	1.89±0.047a

Note: Different letters indicate statistically significant differences ($p < 0.05$) after ANOVA and Duncan's test.

Table S2. Relative expression of ZmRBOHs during osmotic stress.

Treatment		-PEG			+PEG		
		0h Calcium	2h Calcium	10d Calcium	0h Calcium	2h Calcium	10d Calcium
Leaf	*ZmRBOHA*	1.00±0.103c	0.85±0.089c	1.65±0.258b	2.54±0.249a	3.05±0.284a	2.53±0.169a
	ZmRBOHB	1.00±0.031c	0.71±0.031d	1.27±0.112b	1.22±0.044bc	1.17±0.044bc	2.73±0.110a
	ZmRBOHC	1.00±0.005e	1.35±0.085d	1.74±0.037c	1.77±0.035c	2.78±0.148b	5.35±0.141a
	ZmRBOHD	1.00±0.050cd	1.13±0.023c	2.09±0.050b	1.11±0.061c	0.89±0.038d	2.50±0.030a
Root	*ZmRBOHA*	1.00±0.003d	1.13±0.039d	2.24±0.039c	2.22±0.062c	2.53±0.065b	2.90±0.062a
	ZmRBOHB	1.00±0.014c	0.85±0.029d	1.26±0.022b	1.30±0.057b	1.27±0.032b	2.71±0.058a
	ZmRBOHC	1.00±0.001e	0.91±0.022e	1.43±0.082d	2.24±0.067c	2.50±0.058b	3.05±0.028a
	ZmRBOHD	1.00±0.001e	1.01±0.007e	2.04±0.032b	1.27±0.020c	1.14±0.053d	2.58±0.060a

Note: Different letters indicate statistically significant differences ($p < 0.05$) after ANOVA and Duncan's test.

Table S3. Relative expression of antioxidant enzyme during osmotic stress

Treatment		-PEG			+PEG		
		0h Calcium	2h Calcium	10d Calcium	0h Calcium	2h Calcium	10d Calcium
Leaf	*ZmCAT1*	1.00±0.020d	0.93±0.022d	1.29±0.017c	1.77±0.057b	2.34±0.108a	2.42±0.114a
	ZmSOD4	1.00±0.027c	1.01±0.018c	2.52±0.219a	1.78±0.061b	1.89±0.012b	2.75±0.034a
	ZmcAPX	1.00±0.050e	1.08±0.025de	1.94±0.044b	1.44±0.052c	1.21±0.030d	2.08±0.044a
	ZmGR1	1.00±0.022d	0.79±0.033e	1.59±0.023a	1.37±0.033b	1.20±0.013c	1.68±0.105a
Root	*ZmCAT1*	1.00±0.028c	1.95±0.597bc	1.22±0.290bc	1.72±0.717bc	2.75±0.166b	4.75±0.619a
	ZmSOD4	1.00±0.205c	1.49±0.402bc	5.48±0.044a	2.21±0.790bc	2.79±0.158b	5.82±0.621a
	ZmcAPX	1.00±0.015d	1.05±0.031d	1.47±0.053bc	1.37±0.041c	1.60±0.076b	2.57±0.120a
	ZmGR1	1.00±0.036d	1.69±0.075c	2.79±0.136b	1.77±0.074c	2.46±0.214b	4.66±0.348a

Note: Different letters indicate statistically significant differences ($p < 0.05$) after ANOVA and Duncan's test.

Table S4. Relative expression of ZmPIPs during osmotic stress in leaves.

Treatment	-PEG			+PEG		
	0h Calcium	2h Calcium	10d Calcium	0h Calcium	2h Calcium	10d Calcium
ZmPIP1;1	1.00±0.092a	1.00±0.038a	0.83±0.040a	0.45±0.051b	0.98±0.062a	0.89±0.037a
ZmPIP1;2	1.00±0.050b	1.73±0.076a	0.91±0.015b	0.53±0.055c	0.65±0.109c	0.66±0.034c
ZmPIP1;3	1.00±0.214b	1.22±0.229b	1.12±0.062b	1.10±0.048b	2.18±0.113a	1.90±0.056a
ZmPIP1;4	1.00±0.030c	3.09±0.221a	1.05±0.027c	0.71±0.150c	1.85±0.066b	1.93±0.040b
ZmPIP1;5	1.00±0.089ab	1.15±0.208a	1.25±0.038a	0.39±0.030c	0.72±0.057b	0.85±0.033b
ZmPIP2;1	1.00±0.120c	1.62±0.260b	1.75±0.064b	1.92±0.180b	2.90±0.212a	3.01±0.016a
ZmPIP2;2	1.00±0.188c	1.45±0.031b	2.13±0.093a	0.57±0.037d	0.70±0.109cd	2.07±0.035a
ZmPIP2;3	1.00±0.188c	1.45±0.031b	1.87±0.055a	0.57±0.037d	0.70±0.108d	1.50±0.035b
ZmPIP2;4	1.00±0.202c	1.57±0.062b	2.20±0.070a	0.49±0.019d	0.99±0.048c	1.99±0.062a
ZmPIP2;5	1.00±0.062c	1.00±0.085c	1.74±0.057a	0.50±0.026d	0.82±0.048c	1.49±0.054b
ZmPIP2;6	1.00±0.060c	1.84±0.158b	2.47±0.047a	0.65±0.059c	1.80±0.269b	2.15±0.096ab

Note: The relative expression was normalized with -PEG 0h treatment. Different letters indicate statistically significant differences ($p < 0.05$) after ANOVA and Dun-can's test.

Table S5. The primers used in qRT-PCR

Primer name	Primer Sequence（5′-3′）	Purpose
NCED/vp14-QF	GGCTTCCACGGCACCTTCATCACGGGC	Expression level
NCED/vp14-QR	CGGGGAACTGATCTGGGCTCCCTCTGG	Expression level
ABA3-QF	CGGCAGGTGTACTTTGGGCAAA	Expression level
ABA3-QR	CGGGGTCCTGATTCGGTCACTCAG	Expression level
ZmAO2-QF	GGCCTCCTCTCCAGAATACC	Expression level
ZmAO2-QR	ACCTCAACA GCAATGGAACC	Expression level
β-tubulin-QF	GTACCGCGCTCTCACCGTGC	Expression level
β-tubulin-QR	ACGGAACATAGCAGATGCCGTGA	Expression level
Zmactin1-QF	ATCCAGGCTGTTCTTTCGTT	Expression level
Zmactin1-QR	CATTAGGTGGTCGGTGAGGT	Expression level
SOD4-QF	TGCTCGCCTACAAATCCA	Expression level
SOD4-QR	TAGCGTTCTCAAATGCTTAA	Expression level
cAPX-QF	CGACAAGGCCAAGCGTAA	Expression level
cAPX-QR	GCTCCCTCAAATCCAGACC	Expression level
GR1-QF	CATACAGAACAGAGCCCTCA	Expression level
GR1-QR	TTGGCAACGAAGACATCA	Expression level
CAT1-QF	CCAAGGGTTTCTTTGAGGT	Expression level
CAT1-QR	AGGGTCGAAGGAACGATAT	Expression level
ZmSnRK2.2-QF	CGAAGAATTTCCGCAAGACG	Expression level
ZmSnRK2.2-QR	TCGCCACAAAGATCCGAGAC	Expression level
ZmSnRK2.3-QF	CGCCTTCTGTCTCAGATATTCG	Expression level
ZmSnRK2.3-QR	GCATCCGCGTCCTTGTAGTT	Expression level
ZmSnRK2.6-QF	ACATCGGTTCCGGCAACTT	Expression level
ZmSnRK2.6-QR	TCCCTCGCCACATTCTCGT	Expression level
ZmSnRK2.10-QF	AGATCCTGGCCGAGGCGAC	Expression level
ZmSnRK2.10-QR	TACACGATTTCCCCACTGCTTTC	Expression level
ZmPP2CA-QF	GATGCTGGCTCAACTGCCT	Expression level
ZmPP2CA-QR	GAAACCGCAATACCCTGTCC	Expression level
ZmPP2C ABI1-QF	CACATCGTGGTCGCCAACT	Expression level
ZmPP2C ABI1-QR	CCACCTGCTGATTCTACCCTTT	Expression level
ZmPP2C ABI2-QF	AGCACTGGGAGAAGGTCTTTGT	Expression level
ZmPP2C ABI2-QR	GGCAACGATGACATGCGAT	Expression level
ZmrbohA-QF	ATGACATTCTTCTGCTTATTGGC	Expression level
ZmrbohA-QR	ATGCTTCCCACCTCTTCGTT	Expression level
ZmrbohB-QF	ACCCTTTGAATGGCATCCG	Expression level
ZmrbohB-QR	AAGGAGTTGCACCAATCCCTAAT	Expression level
ZmrbohC-QF	GAATACGAAAGCTGCACGGGCATT	Expression level
ZmrbohC-QR	CCAAAGTATTTGCGCAGTGGAGCA	Expression level
ZmrbohD-QF	CCGGCTGCAGACGTTCTT	Expression level
ZmrbohD-QR	CCTGATCCGTGATCTTCGAAA	Expression level

ZmPIP1;1-QF	CCCCTACTAGTTTACGTGGAGTTC	Expression level
ZmPIP1;1-QR	GCGGCATATTACACAATTGGTA	Expression level
ZmPIP1;2-QF	CTCATTTTATGCGTTGGGATGT	Expression level
ZmPIP1;2-QR	ACTGAAACAAGAAAACCCTGA	Expression level
ZmPIP1;3-QF	GGTTCCCGTATCCTTTTATGC	Expression level
ZmPIP1;3-QR	AATCCAGCTGATAGATAAACCCAC	Expression level
ZmPIP1;4-QF	GCCATCTACCACCAGGTGAT	Expression level
ZmPIP1;4-QR	GGGCAGACAATACATTCCCC	Expression level
ZmPIP1;5-QF	CACGTGGTCATCATCAGGG	Expression level
ZmPIP1;5-QR	CGTATGCTGCATGGTTGCT	Expression level
ZmPIP2;1-QF	CGGGTCGCCTTTTTTTTG	Expression level
ZmPIP2;1-QR	CCCTTGAGAGTCACGACATGA	Expression level
ZmPIP2;2-QF	GGCCTTCTACCACCAGTACATC	Expression level
ZmPIP2;2-QR	GGCCTTTCTTTAGCTCTGCTC	Expression level
ZmPIP2;3-QF	AGTACGTGCTGAGAGCCAGC	Expression level
ZmPIP2;3-QR	CGTACGTATCTACACTTGGATCGAT	Expression level
ZmPIP2;4-QF	TACCGGAGCAACGCCTAAG	Expression level
ZmPIP2;4-QR	GAAAACAGCAGCGAGCGA	Expression level
ZmPIP2;5-QF	TGTCGTCGTTGGTTGCCT	Expression level
ZmPIP2;5-QR	CACAACAATCACACTAGCTTGGAA	Expression level
ZmPIP2;6-QF	TTTAAGGTGAACGGAGAAGGAGA	Expression level
ZmPIP2;6-QR	GAAAGCTACTGCTGCTGTGGAT	Expression level
